# Instruments for the care of pressure injury in pediatrics and hebiatrics: an integrative review of the literature

**DOI:** 10.1590/1518-8345.2289.3034

**Published:** 2018-08-09

**Authors:** Mayara Kelly Moura Ferreira, Sabrina de Souza Gurgel, Francisca Elisângela Teixeira Lima, Maria Vera Lúcia Moreira Leitão Cardoso, Viviane Martins da Silva

**Affiliations:** 1 Master’s student, Universidade Federal do Ceará, Fortaleza, CE, Brazil. Scholarship holder at Coordenação de Aperfeiçoamento de Pessoal de Nível Superior (CAPES), Brazil.; 2 PhD, Adjunct Professor, Departamento de Enfermagem, Universidade Federal do Ceará, Fortaleza, CE, Brazil.; 3 PhD, Full Professor, Departamento de Enfermagem, Universidade Federal do Ceará, Fortaleza, CE, Brazil.

**Keywords:** Pressure Ulcer, Pediatrics, Adolescent, Protocols, Technology, Review

## Abstract

**Objective:**

to identify existing instruments in the scientific literature about the care of pressure injuries in pediatric and hebiatric patients.

**Method:**

an integrative review that selected 32 articles from the PubMed/Medline, LILACS, Scopus and CINAHL databases using the descriptors *pressure ulcer, decubitus ulcer, pediatrics,* *adolescent* and *protocols* and their equivalent terms in Portuguese and Spanish. Inclusion criteria: research articles in full, in Portuguese, English or Spanish without any temporal cut-off. The descriptive analysis was adopted to categorize the instruments found into the three domains of care: evaluative, preventive and curative.

**Results:**

25 instruments for the care of pressure injuries in pediatric and hebiatric patients were found, with an emphasis on the Braden Q Scale being the most commonly used to assess pressure injury risk, and the protocols containing pressure injury staging guidelines developed by the *National Pressure Ulcer Advisory Panel* (NPUAP) and the *European Pressure Ulcer Advisory Panel* (EPUAP).

**Conclusion:**

the scientific literature has valid and reliable instruments that can be used in nursing care practice aimed at the evaluative, preventive and curative care of pressure injuries in pediatric and hebiatric patients.

## Introduction

The care for hospitalized children and adolescents requires the nursing team to recognize their needs, taking into account the different stages of development^1^. To exercise this care, the use of technologies that cover the technical and scientific knowledge translated into tools, processes and materials created and used is essential^2^, systematizing the care to favor the maintenance of life, provide comfort, well-being and recovery of health.

Among these technologies, we can highlight instruments that express systematized thinking with a specific purpose, which represents a record/registry and promotes and guides the care process. They can be represented as a consultation script, a scale, a functional organization chart, a protocol, a checklist, or a questionnaire, among others^3^. They represent tools that qualify the care and reflect the care quality 

Indicators have been studied in seeking care quality, benefiting hospitalized patients^4^; among these, we can highlight pressure injuries, which are part of the set of quality indicators related to nursing care processes^5^. The term “*pressure injury”* has been used to replace the terminology of *pressure ulcer* previously used by health professionals, a change that took place in April 2016^6^. Although they are more frequent in the older adult and adult population, pediatric patients are also at risk of developing these injuries.

Studying pressure injuries is important, since prevention and treatment are attributed as a way to reduce patients hospital stay and thus diminish costs, prevent infections and improve patient prognosis^7^. 

Data collected regarding the prevalence of pressure injuries in the United States in hospitalized patients aged 0 to 18 years showed prevalence rates of 1.4% and prevalence of hospital-acquired pressure injuries of 1.1%. Rates are even higher among patients in pediatric intensive care units (3.7%) and pediatric rehabilitation (4.6%), while the general pediatric units had a lower prevalence (0.57%)^8^. In Brazil, the prevalence of pressure injuries among children and adolescents at a hospital in Paraná was 8.06%^9^. In a pediatric intensive care unit in Ceará, seventeen children (42.5%) developed 26 pressure injuries^10^. 

In this context, nursing professionals use care technologies that include knowledge justified by scientific principles and proposals of techniques, procedures and knowledge for their care practice^2^. 

Based on the risk of developing pressure injuries in pediatric and hebiatric patients, the use of instruments so that specific interventions are incorporated into patient care is urgent^11^, which may characterize it as care technology. Preventing pressure injury is a nursing responsibility, whose instruments are important components for effective and efficient care^12^. It is noticeable that these instruments are increasingly present in the routine of nursing professionals, becoming indispensable care tools in pediatric and hebiatric medicine.

In view of the above, we consider it is fundamental to promote the discussion about the instruments used in the care of pressure injuries in pediatric and hebiatric patients. 

Considering the importance that instruments which focus on the care of pressure injuries in this population have for nursing professionals, an integrative review was carried out with the objective to identify existing instruments in the scientific literature about the care of pressure injuries in pediatric and hebiatric patients.

## Methods

This is an integrative review of literature in the field of pediatrics and hebiatrics on instruments for the care of pressure injuries. Six steps were followed for this review: identifying the theme and selecting the hypothesis or research question to elaborate the integrative review; establishing criteria for inclusion and exclusion of studies/sampling or search in the literature; defining the information to be extracted from the selected studies/categorization of the studies; evaluating the studies included in the integrative review; interpreting the results; and presenting a knowledge review/synthesis^13^.

The searches were conducted from October to December 2016, guided by the following question: which instruments exist in the scientific literature for the care of pressure injuries in pediatric and hebiatric patients? 

The following databases were used: National Library of Medicine and National Institutes of Health (PubMed/Medline); Latin American and Caribbean Center on Health Sciencies Information (LILACS); SCOPUS and Cumulative Index to Nursing and Allied Health Literature (CINAHL), based on the descriptors of the Health Sciences Descriptors (DeCS): pressure ulcer, decubitus ulcer, pediatrics and protocols, and their equivalents in English/Portuguese in the *Medical Subjects Headings* (MESH). For this procedure, two independent researchers simultaneously performed the collection by combining the search terms in each database. The searches at SCOPUS, PubMed/Medline and CINAHL databases were initially performed in relation to pediatrics, according to the following crosses: *pressure ulcer* AND *pediatrics* AND *protocols*; *decubitus* *ulcer* AND *pediatrics* AND *protocols*; *pressure* *ulcer* AND *pediatrics*; *decubitus* *ulcer* AND *pediatrics*; *pressure ulcer* AND *protocols*; e *decubitus ulcer* AND *protocols*. Although pediatrics is defined in the DeCS and MESH as a specialty aimed at maintaining health and providing care to children from birth to adolescence, the databases were also searched in relation to hebiatrics using the crosses: *pressure ulcer* AND *adolescent* AND *protocols; decubitus* *ulcer* AND *adolescent* AND *protocols*. In addition to the English equations in the LILACS database, variations of the descriptors in Spanish and Portuguese were used. 

We chose the age group between 0 to 19 years according to the World Health Organization (WHO) classification, which considers those up to nine years of age as children, and those in the chronological limit between 10 and 19 years as adolescents^14^.

The inclusion criteria were: answering the guiding question; constituting a research article available in full in Portuguese, English or Spanish with no temporal cut-off; published in scientific journals and available electronically.

The following exclusion criteria were adopted: literature review, editorials, reviews, case reports, case studies, theoretical reflections, dissertations, theses, monographs and abstracts published in event archives. Repeated articles were also excluded, thus only maintaining the first identified version. 

**Figure 1:**
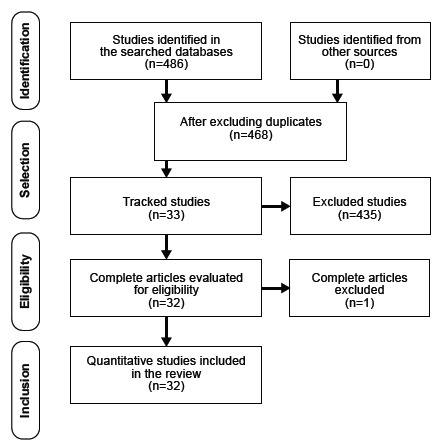
Flowchart of the study identification, selection and inclusion process based on the PRISMA recommendations^15^. Fortaleza, CE, Brazil, 2016.

An instrument prepared by the authors was used in order to collect the data, in which the necessary information to answer the research question such as titles, authors, years, bases, objectives, environments, populations and instruments used were organized. In addition to the data characterizing the studies, we sought to extract information about the type of instrument (scale, protocol, *checklist*, questionnaire, computerized system and form), the population and the application environment of the instruments. The study selection followed the recommendations of the Preferred Reporting Items for Systematic Reviews and Meta-Analyses (PRISMA)^15^, as shown in Figure 1. The articles initially selected in a consensual way (by the independent researchers) were automatically accepted. The articles selected in a discordant manner were discussed on a case-by-case basis until agreement was reached. 

The descriptive analysis followed the following steps: reading the articles in full; categorizing the instruments into three areas of care: evaluative, preventive and curative; and discussing the articles according to the categories. Some instruments belonged to more than one domain.

Evaluative care in this study was established as care that evaluates the risk of developing a pressure injury through predictive instruments and installed lesions regarding their characteristics; preventive care was defined as the implementation of measures aimed at preventing the onset of pressure injury; and curative care refers to the treatment of pressure injuries^16^.

No qualitative articles were selected. The quantitative articles found in the investigated databases and included in this review are presented in Table 1.

**Table 1:** Quantitative articles found in the databases and included in the study. Fortaleza, CE, Brazil, 2016.

Database	Interface	Found with the pediatric descriptors*	Found with the hebiatric descriptors*	Included*
MEDLINE	*National Center for Biotechnology Information* ncbi.nlm.nih.gov/pubmed	147	15	15
LILACS	*Biblioteca Virtual de Saúde* - BVS bvsalud.com	7	8	0
CINAHL	EbscoHost web.a.ebscohost.com	191	0	13
SCOPUS	Scopus.com	75	43	4
Total		420	66	32

* Some publications were found in more than one database.

## Results

Twenty-five (25) instruments for the care of pressure injuries in pediatrics and hebiatrics were identified from the 32 articles of this review. Of these, 13 referred to evaluative care, five to evaluative/preventive care, four to preventive care, one to curative care, one to preventive/curative care and one to evaluative/preventive/curative care. 

The articles highlighted the validity and reliability of seven instruments: *Braden Scale,* *Braden Q Scale*, *Braden Q+P Scale*, *Glamorgan Scale*, *Garvin Scale*, *Pediatric Pressure Ulcer and Evaluation Tool* and the *Starkid Skin Scale*. The articles did not include information about the validation process regarding other instruments. 

The Pediatric Intensive Care Unit (PICU) was highlighted regarding the application environment of these instruments with 17 articles. Regarding the population, 20 articles contemplated hebiatrics. 

Among the selected articles, most of them were published in 2013 (seven articles), with a decrease in the following years for 2014 (three articles) and 2015 (three articles), while five articles were identified in 2012. Two articles were published in the following years: 2011, 2010, 2009 and 2003. The years with the least number of publications were: 2007, 2006, 2005, 2002, 1996, 1994, with only one article identified each year. No publications on the subject were identified in the years 2016, 2008, 2004, 2001, 2000, 1999 and 1995. 

The main language of dissemination was English (30 articles), followed by those published in Portuguese (two articles). The predominance of English demonstrates its adoption as the main language of dissemination established by several journals in the world. However, this preponderance does not indicate that the selected articles were produced in countries with that language. Most articles were developed in the United States (16 articles), Australia, England, Brazil and Switzerland (two articles each), Spain, India, Nigeria, Jordan, Canada, Germany, China and Wales (one article each). 

**Figure 2:**
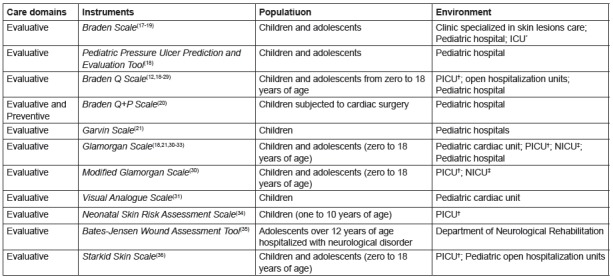
List of the instruments and scale type for the care of pressure injuries in pediatrics and hebiatrics. Fortaleza, CE, Brazil, 2016.

Three figures were organized according to the types of instruments found in order to facilitate comprehension. Figure 2 shows the 11 scales found.

*ICU - Intensive Care Unit; †PICU - Pediatric Intensive Care Unit; ‡ NICU - Neonatal Intensive Care Unit

According to Figure 2, among the scales related to pressure injury, eleven had an evaluative character and one also addressed the preventive aspect, which included those used to assess the risk of lesion development and also those to evaluate the evolution of installed lesions. Most of the scales were applied both in pediatrics and hebiatrics; however, four were only focused on pediatrics. The predominant environment was the hospital, with an emphasis on critical units.

**Figure 3:**
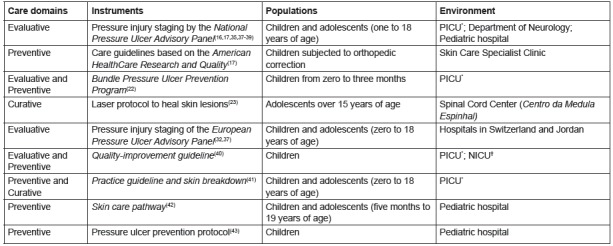
List of instruments and protocol type for the care of pressure injury in pediatrics and hebiatrics. Fortaleza, CE, Brazil, 2016.

Figure 3 shows the nine protocols found in the review.

*PICU - Pediatric Intensive Care Unit

†NICU - Neonatal Intensive Care Unit

Regarding the protocols (Figure 3), a predominance of those in the preventive domain were found, differing from the scales in which the evaluative domain was more prominent. Similarly to the scales, most of the protocols were applied in pediatrics and hebiatrics; however, four were directed only to pediatrics. The hospital environment prevailed, with a focus on the critical units. 

**Figure 4:**
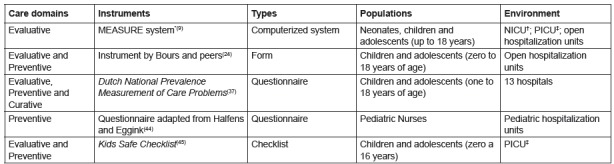
List of other instruments for the care of pressure injury in pediatrics and hebiatrics. Fortaleza, CE, Brazil, 2016.

Figure 4 presents the other instruments found, two questionnaires, one system, one form and a checklist.

* MEASURE - stands for: M (measure); E (exudate); A (appearance); S (suffering); U (undermining); R (re-evaluation); E (edge)

†NICU - Neonatal Intensive Care Unit

‡ PICU - Pediatric Intensive Care Unit

According to Figure 4, a predominance of evaluative and preventive domains was observed among the other instruments. Four of them were aimed at children and adolescents, and one had pediatric nurses as its target population. Regarding the application environment, critical and the open hospitalization units can be highlighted. 

## Discussion

In the search for instruments aimed at the care of pressure injuries in pediatrics and hebiatrics, we have identified predictive scales for evaluating the risk of developing lesions, and evaluation scales for the characteristics of installed lesions, protocols and other instruments in the three care domains: evaluative, preventive and curative. 

The risk for pressure injury can be identified by applying the scales. The oldest publication of this review using the Braden Scale dates back to 2003^17^. This scale was created in 1987, and it evaluates and accounts for etiological factors that contribute to reducing tissue tolerance to prolonged compression through six subscales: sensory perception; moisture; activity; mobility; nutrition; and friction and shear. Three of them measure clinical determinants of exposure to intense and prolonged pressure - sensory perception, activity and mobility; and three measure tissue tolerance to pressure - moisture, nutrition, friction and shear. The first five subscales are scored from one to four; the sixth is scored from one to three. The score can be classified considering values ≤ 9 as very high risk; 10 to 12 as high risk; 13 to 14 as moderate risk; 15 to 18 as low risk; and values from 19 to 23 as no risk for the development of pressure injury^46^. 

The Braden Scale was adapted for the pediatric population and entitled the *Braden Q Scale.* It was created in 1996, constituting as a tool specifically for pediatric pressure ulcer risk assessment^47^. The oldest publication using this scale dates from the same year of its origin^43^. This scale includes the six original subscales of the *Braden Scale*, in addition to “tissue perfusion and oxygenation” as the seventh subscale, reflecting that scale modifications reflect unique development characteristics of pediatric patients. Patients only receive one score per subscale, which is scored from 1 (least favorable) to 4 (most favorable). The total Braden Q scale ranges from 7 (highest risk) to 28 (lower risk), where scores below or equal to 16 identify pediatric patients at risk of developing pressure injury^47^. 

The Braden Q Scale has also been modified and adapted, constituting the Braden Q + P Scale, which is targeted at pediatric patients undergoing cardiac surgery as part of a comprehensive pressure injury prevention initiative that guides the planning of nursing interventions^20^. 

Other scales were also found in the review, among them the Glamorgan Scale, designed directly for children and adolescents. It consists of nine items: mobility, pressure equipment, anemia, pyrexia, peripheral perfusion, nutrition, serum albumin, weight and incontinence. The mobility item consists of four categories that can be classified as 0 (normal mobility for age), 10 (some mobility, but reduced for age), 15 (unable to change position without assistance/cannot control body movement), and 20 (child cannot be moved without great difficulty). The equipment/objects/hard surface pressing or rubbing on skin item can be classified as 0 (no) or 15 (yes) points. The items significant anemia (Hb < 9g/dl), persistent pyrexia (temperature > 38°C for more than 4h), poor peripheral perfusion (cold extremities/ capillary refill > 2 seconds/cool mottled skin), inadequate nutrition, low serum albumin (<35 g/l), weight less than the 10^th^ percentile, and Incontinence (inappropriate for age) are classified as 0 or 1. The scores of the items are summarized in scores resulting between zero and 42. The higher the score sum, the higher the risk of pressure injury^48^.

The Glamorgan Scale has been modified (mGS), and even though it is simpler in terms of usability and efficiency, it has been observed that this tool is equally effective in categorizing patients as the original Glamorgan Scale, producing the same categorization of risk in the assessment of pediatric patients^30^.

By using the area under the ROC curve to compare the Braden Q Scale (area of 0.82, p<0.001, 95% CI=0.760-0.880) with the Glamorgan Scale (area of 0.748, p<0.001, 95% CI=0.673-0.822), it was observed that both have the same predictive power since the confidence intervals overlapped. Both scales probably have similar validity in the Pediatric Intensive Care Unit (Glamorgan: area of 0.76, p=0.006, 95% CI=0.61-0.91; Braden Q: area of 0.74, p=0.010, 95% CI=0.58-0.90) and in the Neonatal Intensive Care Unit (Glamorgan: area of 0.82, p<0.001, 95% CI=0.73-0.91; Braden Q: area of 0.82, p<0.001, 95% CI=0.73-0.92) and for more serious injuries, although the Braden Q has shown better results in pediatric wards (Glamorgan: area of 0.57, p=0.478, 95% CI=0.37-0.77; Braden Q: area of 0.83, p<0.001, 95% CI=0.73-0.92)^49^.

In addition to these scales, the Garvin Scale is based on four risk factors: mobility, sensory perception, nutrition and moisture, in which a total score is used to divide children into categories with increasingly intense interventions as the risk increases^21^. 

The comparison between the Glamorgan Scale, the Braden Q Scale, and the Garvin Scale identified that the Glamorgan Scale (area of 0.912, p<0.001, 95% CI=0.873-0.946) has a greater predictive power than the Braden Q scale (area of 0.694, p<0.001, 95% CI=0.627-0.762) or the Garvin scale (area of 0.641, p=0.001, 95% CI=0.570-0.712)^21^. Nevertheless, we can point out that the Braden Q Scale is the most used in studies, possibly because it was the first to be published. 

Other assessment scales were found addressing skin ruptures in neonates (*Neonatal Skin Risk Assessment Scale* - NSRA)^34^, in which the degree of risk perceived by the nurses is marked on a 100 mm horizontal line, anchored by word descriptors at each end such as “no risk” and “maximum risk” (*Visual Analogue Scale*-VAS)^31^; and also a scale that measures the risk of skin rupture with a high level of specificity (*Starkid Skin Scale*)^36^.

In addition to these, the *Bates-Jensen Wound Assessment Tool* (BWAT) was also found, which differs from the others as it evaluates installed injuries by considering the following aspects: wound size, depth, edges, undermining, type of necrotic tissue, amount of necrotic tissue, granulation tissue, epithelialization (tissue), type of exudate, amount of exudate, surrounding skin, peripheral edema and hardening of the peripheral tissue. This tool has 13 items that use a modified Likert scale in which a score of 1 indicates the healthiest, and 5 the unhealthiest attribute for each trait. The total score of the scale is obtained by adding the individual scores of each evaluation item, with the total value ranging from 13 to 65^35^. It was translated and adapted for Brazilian culture in 2015, and named the *Bates-Jensen Wound Assessment Tool* - Brazilian version^50^. 

The *Pressure Ulcer Prediction and Evaluation* *Tool* (PPUPET) is a scale that evaluates aspects related to mobility, activity, sensory perception, humidity, external medical devices, friction/shear, perfusion and tissue oxygenation, skin condition and nutrition^18^.

In view of the above, nurses could more accurately determine interventions for prevention and treatment of pressure injury by applying a predictive risk scale and performing a clinical evaluation, then using this data to conduct a care plan for pressure injuries^51^. 

Care plans can follow existing guidelines and protocols. Some publications have adopted the guidelines for pressure injury staging developed by the *National Pressure Ulcer Advisory Panel* (NPUAP) and the *European Pressure Ulcer Advisory Panel* (EPUAP), which are recommendations based on evidence for evaluating pressure injuries to be used by health professionals worldwide, helping to choose interventions related to pressure injury^52^. In addition, there are care guidelines based on the *American Health Care Research and Quality* (AHRQ), which has the mission of producing evidence to make health care safer, affordable, equitable and of higher quality^17^. There is also the *Quality-improvement guideline*, which is focused on reducing the rate of pressure injury in pediatric and neonatal intensive care units^40^. With a similar proposal, the *Practice guideline and skin breakdown* was created to direct the practice and guide the professionals of pediatric cardiac intensive care units about interventions for possible problems of skin rupture in patients^41^.

Protocols for the prevention and/or treatment of pressure injury are also part of existing instruments for such purposes. The *Bundle Pressure Ulcer Prevention Program* (PUPP) was evaluated to determine whether there is an association between its implementation and a significant reduction in the development of pressure injury in a pediatric intensive care unit^22^. 

The *Skin care pathway* was developed for standardizing care aimed at preventing pressure injury complications arising from orthopedic corrections in children and adolescents^42^. 

Another protocol sought to compare the effect on healing when nursing care was received alone by adolescents older than 15 years of age with spinal cord injury who had pressure injury, with the implication on wound healing in nursing care combined with laser treatment or with an ultrasound/ultraviolet C. The results demonstrated that they can shorten the healing time, which allows a faster return to rehabilitation programs, to work and leisure activities for patients with spinal cord injury who have pressure injury^23^. 

In this review, we have identified a protocol for preventing pressure injury developed in 1996, which sought to provide nurses with tools to assist them in making clinical decisions regarding pediatric patients with potential or actual changes in their skin integrity. The components of this protocol included a risk assessment tool for the development of pressure injury (Braden Q), a skin care algorithm to decrease unnecessary variation in preventive care practices and a pressure injury algorithm to reduce unnecessary variation in staging and management of patients with pressure injury^43^.

Another questionnaire type instrument found was the *Dutch National Prevalence Measurement of Care Problems* (LPZ), which includes the following categories for evaluation: patient characteristics (demographic and clinical data); assessment of the location and severity of pressure injuries; assessment of the risk of pressure injury according to the Braden Scale; predisposing factors for the development of pressure injury; preventive interventions and therapeutic interventions^37^. 

An instrument developed by Bours et al. was used to assess prevalence, severity, risk factors, and preventive and therapeutic interventions for pressure injuries. This includes six data categories to be collected: characteristics of the health facilities, the ward or the staff, from the patients (demographic and clinical data), an evaluation of the risk of developing pressure injury using the Braden scale, assessment of the severity of pressure injuries and preventive interventions implemented^24^. 

One of the publications in this review^9^ used to evaluate neonates, children and adolescents implemented an instrument composed of patient characteristics related to sociodemographic and clinical data, anatomical location and clinical evaluation of the wound, and the MEASURE mnemonic which stands for: M (measure); E (exudate); A (appearance); S (suffering); U (undermining); R (re-evaluation); E (edge), created in 2004, which includes the classification of pressure injuries in relation to its Measure (length, width, depth and area) Exudate (quantity and quality), Appearance (wound bed, including tissue type and amount), Suffering (type and level of pain), Undermining (presence or absence), Reevaluation (monitoring of all parameters regularly), and Edge (condition of edge and surrounding skin)^53^.

Another instrument was developed to undertake holistic pediatric care, incorporating the physiological and developmental requirements of children and adolescents, focusing on preventable adverse events^45^ which consists of a checklist (*Kids safe checklist*) comprising eight care areas for patients in pediatric intensive care units: developmental needs, infection, prophylaxis of deep venous thrombosis, skin integrity, sedation, analgesia and enteric needs. This checklist incorporates important priorities for members of the multidisciplinary team and can be easily adapted to local needs^45^. Checklists are used throughout health care to ensure adherence to key processes for safer and more reliable care^54^. 

In general, the instruments were aimed at children and adolescents; however it can be questioned whether those instruments aimed only at pediatrics could not be adapted and used for hebiatric/adolescent patients, since both groups are at risk of developing pressure injuries. 

As a contribution to clinical practice, this integrative review grouped and synthesized knowledge on instruments for the care of pressure injuries in pediatrics and hebiatrics with the purpose of making evidence-based practice of health professionals feasible in the care of children and adolescents.

As a limitation we can point out the methodological data collection from the studies, considering that many did not explicitly present a description of the study type, making it difficult to identify the levels of evidence. 

## Conclusion

The results obtained in this review show 25 existing instruments in the literature that can be used for the care of pressure injuries in pediatric and hebiatric patients, including scales, protocols, system, forms, checklists and questionnaires. We point out the Braden Q scale was the most used instrument to assess the risk of pressure injury. With regard to protocols, we can point out adopting the guidelines for the staging of pressure injuries developed by the NPUAP and the EPUAP. 

Although pressure injury is not very common in this population, the results have contributed to show that the subject has been studied and that the instruments for evaluative care have been most used to provide qualified and safe care in pediatrics and hebiatrics. 

In view of the above, nurses have instruments available to evaluate, prevent and treat pressure injuries, which will assist in creating and implementing a specific care plan based on scientific evidence. 

Skin care is one of many nurses’ roles, however these professionals must work together with the multiprofessional team to assess, prescribe and implement preventive and curative care of pressure injuries by making use of the technologies in this process.
